# Visceral fat metabolic activity evaluated by preoperative ^18^F-FDG PET/CT significantly affects axillary lymph node metastasis in postmenopausal luminal breast cancer

**DOI:** 10.1038/s41598-020-57937-4

**Published:** 2020-01-28

**Authors:** Kisoo Pahk, Chanmin Joung, Sungeun Kim

**Affiliations:** 1 Department of Nuclear Medicine, Korea University Anam Hospital, Seoul, Republic of Korea; 2 Institute for Inflammation Control, Korea University, Seoul, Republic of Korea

**Keywords:** Prognostic markers, Breast cancer

## Abstract

Obesity is known to increase breast cancer risk and aggressiveness in postmenopausal luminal breast cancer and obesity-driven dysfunctional metabolic activity in visceral adipose tissue (VAT) is considered as one of the principal underlying mechanism. We aimed to investigate the relationship between VAT metabolic activity evaluated by preoperative ^18^F-FDG PET/CT and axillary lymph node (ALN) metastasis in postmenopausal luminal breast cancer patients. In total, 173 patients were enrolled in study. They all underwent preoperative ^18^F-FDG PET/CT and surgery. VAT metabolic activity was defined as the maximum standardized uptake value (SUVmax) of VAT divided by the SUVmax of subcutaneous adipose tissue (V/S ratio). In luminal breast cancer, the patients with ALN metastasis showed significantly higher V/S ratio than the patients without ALN metastasis. Furthermore, V/S ratio was significantly associated with ALN metastasis in luminal breast cancer patients. Erythrocyte sedimentation rate, which reflect the systemic inflammation, was significantly higher in ALN metastasis group than the negative ALN metastasis group in luminal breast cancer patients and showed significant positive correlation with V/S ratio. V/S ratio significantly affects the ALN metastasis status in postmenopausal luminal breast cancer patients and it may be useful as a potential biomarker of obesity-driven systemic inflammation associated with tumor aggressiveness.

## Introduction

Obesity is a major public health problem today that affecting over 600 million adults worldwide and more than 36% of adults in the United States^1,2^. The prevalence of obesity is continuously increasing throughout the world including both developed and developing countries^3^.

Up to date, accumulating evidences of randomized clinical trials and meta-analyses support that obesity increases breast cancer risk and cancer aggressiveness, which lead to increase mortality and morbidity, in postmenopausal luminal breast cancer^4,5^. One of the key pathophysiological mechanism underlying obesity and postmenopausal luminal breast cancer is related to the dysfunctional visceral adipose tissue (VAT) that promotes tumorigenesis and metastasis^5,6^. In postmenopausal women, estrogen synthesis is largely catalyzed in adipose tissue, through the conversion of androgens to estrogens by aromatase^7^. Dysfunctional VAT secretes pro-inflammatory cytokines such as tumor necrosis factor-alpha (TNF-α) and interleukin-6 (IL-6), thereby stimulating aromatase activity, which eventually drives higher estrogen production to support luminal type breast cancer growth^5,6^. Furthermore, pro-inflammatory cytokines secreted by dysfunctional VAT also promotes infiltrating tumor-associated macrophages, upregulates cancer stem cells, and stimulates tumor angiogenesis, which contribute to create a pro-oncogenic environment^5,6^.

Axillary lymph node (ALN) metastasis has been regarded as one of the most important factors for choosing optimized treatment option and determining the prognostic outcome in patients with breast cancer^8,9^. Previous epidemiologic studies report that obesity is associated with positive ALN metastasis in postmenopausal breast cancer patients^10,11^. However, little is known about the metabolic activity of dysfunctional VAT and ALN metastasis in postmenopausal luminal breast cancer.

^18^F-fluorodeoxyglucose positron emission tomography/computed tomography (^18^F-FDG PET/CT) is a well-established non-invasive imaging tool for evaluation of VAT metabolic activity^12–15^. Increased activity of classically activated (M1) macrophage plays pivotal roles in dysfunctional VAT-driven systemic inflammation^16^ and ^18^F-FDG PET/CT could reflect the activity of M1 macrophage^15^. Furthermore, recently, increased VAT metabolic activity assessed by ^18^F-FDG PET/CT has been associated with regional lymph node metastasis in thyroid cancer and distant metastasis in colorectal cancer, for which obesity is a risk factor^14,15^. Thus, we hypothesized that increased VAT metabolic activity might also affect the ALN metastasis in postmenopausal luminal breast cancer patients.

The aim of this study was to investigate the relationship between VAT metabolic activity assessed by ^18^F-FDG PET/CT and the status of ALN metastasis in postmenopausal luminal breast cancer patients.

## Results

Of the 173 breast cancer patients, 65 patients were confirmed as ALN metastasis, 108 as negative ALN metastasis. ALN metastasis group showed significantly higher tumor SUVmax, VAT SUVmax, and V/S ratio than negative ALN metastasis group. The clinical characteristics of enrolled patients were summarized in Table 1.

**Table 1:** Patient characteristics.

	ALN metastasis (−)	ALN metastasis (+)	*p*
No. of patients	108	65	
Age (years)	62.1 ± 8.7	60.6 ± 7.2	0.246
BMI (kg/m^2^)	24 ± 3.5	25.5 ± 4.1	0.021*
≥25, n (%)	40 (37)	34 (52.3)	0.049*
<25, n (%)	68 (63)	31 (47.7)	
Histologic grade, n (%)			0.353
1	32 (29.6)	14 (21.5)	
2	42 (38.9)	32 (49.2)	
3	34 (31.5)	19 (29.3)	
Ki-67 status, n (%)			0.142
Low (<20%)	66 (61.1)	47 (72.3)	
High (≥20%)	42 (38.9)	18 (27.7)	
Tumor size (cm), median (IQR)	1.5 (0.9–2.1)	2.3 (1.6–3.5)	<0.001*
Pathologic T stage, n (%)			<0.001*
T1	81 (75)	26 (40)	
T2	27 (25)	31 (47.7)	
T3	0	7 (10.8)	
T4	0	1 (1.5)	
Histology, n (%)			0.442
Ductal	92 (85.2)	56 (86.2)	
Lobular	4 (3.7)	4 (6.2)	
Other	12 (11.1)	5 (7.6)	
Lymphovascular invasion, n (%)			<0.001*
Negative	98 (90.7)	40 (61.5)	
Positive	10 (9.3)	25 (38.5)	
Molecular subtypes, n (%)			0.037*
Luminal A	45 (41.7)	33 (50.7)	
Luminal B	29 (26.9)	24 (36.9)	
HER2-overexpression	13 (12)	4 (6.2)	
Triple-negative	21 (19.4)	4 (6.2)	
ESR, median (IQR)	13.5 (8–19.5)	17 (11–24)	0.015*
Metabolic parameters			
Tumor SUVmax, median (IQR)	2.35 (1.69–3.77)	3.17 (1.95–5.92)	0.012*
VAT SUVmax, median (IQR)	0.36 (0.3–0.4)	0.39 (0.34–0.45)	0.009*
SAT SUVmax, median (IQR)	0.23 (0.2–0.26)	0.22 (0.19–0.26)	0.732
V/S ratio, median (IQR)	1.56 (1.34–1.76)	1.65 (1.51–2.06)	<0.001*
Type of surgery, n (%)			0.046*
Breast-conserving surgery	79 (73.1)	38 (58.5)	
Mastectomy	29 (26.9)	27 (41.5)	
Hormone replacement therapy, n (%)			0.522
None	89 (82.4)	51 (78.5)	
Current user	19 (17.6)	14 (21.5)	

*ALN* axillary lymph node, *BMI* body mass index, *IQR* interquartile range, *HER2* human epidermal growth factor receptor 2, *ESR* erythrocyte sedimentation rate, *SUVmax* maximum standardized uptake value, *VAT* visceral adipose tissue, *SAT* subcutaneous adipose tissue, *V/S ratio* VAT SUVmax/SAT SUVmax.

*Statistically significant difference. *p*-values of Age and BMI were determined by using Student’s *t*-test. *p*-values of Tumor size, ESR, Tumor SUVmax, VAT SUVmax, SAT SUVmax, and V/S ratio were determined by using Mann-Whitney *U* test.

### VAT metabolic activity and body mass index (BMI) in postmenopausal breast cancer patients

As shown in Table 1, the ALN metastasis group showed significantly higher BMI and had a high percentage of patients with increased BMI (≥25) than negative ALN metastasis group. However, in whole patients, there was no significant difference between the VAT SUVmax, SAT SUVmax, and V/S ratio of higher BMI (≥25) and lower BMI group (<25) (0.38 ± 0.08 vs. 0.37 ± 0.08, *p* = 0.508; 0.24 ± 0.06 vs. 0.23 ± 0.05, *p* = 0.179; 1.61 ± 0.3 vs. 1.66 ± 0.37, *p* = 0.517, respectively). In subgroup analysis, there was also no significant difference between the VAT SUVmax, SAT SUVmax, and V/S ratio of higher BMI and lower BMI group in luminal patients (0.38 ± 0.08 vs. 0.38 ± 0.09, *p* = 0.588; 0.24 ± 0.06 vs. 0.23 ± 0.06, *p* = 0.146; 1.61 ± 0.3 vs. 1.67 ± 0.38, *p* = 0.396, respectively) and non-luminal patients (0.35 ± 0.07 vs. 0.36 ± 0.06, *p* = 0.802; 0.22 ± 0.02 vs. 0.23 ± 0.04, *p* = 0.944; 1.61 ± 0.35 vs. 1.62 ± 0.34, *p* = 0.867, respectively).

### VAT metabolic activity is increased in luminal breast cancer patients with ALN metastasis

**Figure 1:**
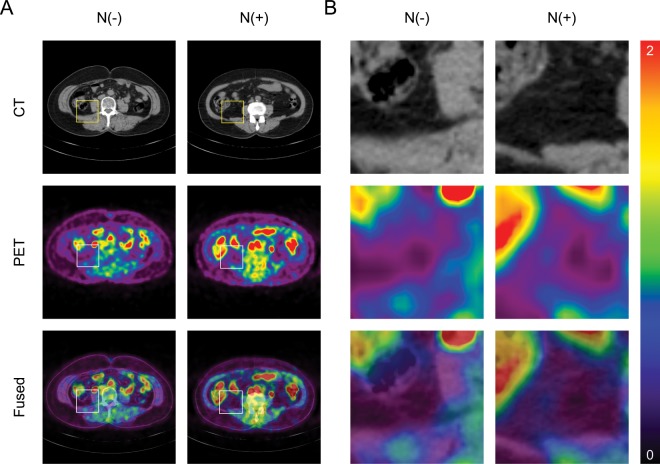
Representative images of visceral adipose tissue (VAT) metabolic activity according to the axillary lymph node (ALN) metastasis status (**A**) and its corresponding magnified VAT images (**B**). N(−); negative ALN metastasis, N(+); positive ALN metastasis, CT; computed tomography, PET: positron emission tomography.

**Figure 2:**
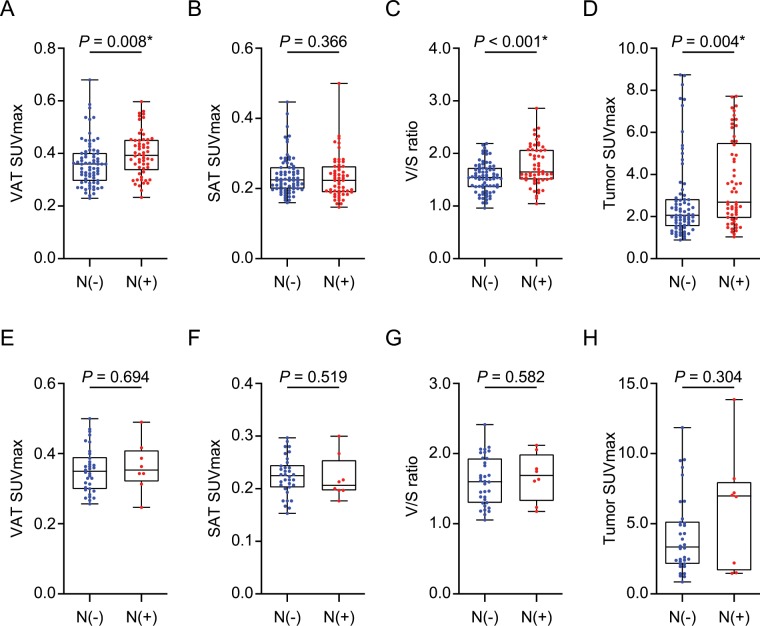
Comparison of (**A,E**) VAT SUVmax, (**B,F**) SAT SUVmax, (**C,G**) V/S ratio, and (**D,H**) Tumor SUVmax according to the ALN metastasis status in postmenopausal luminal and non-luminal breast cancer patients. Luminal breast cancer (**A–D**). N(−), *n* = 74; N(+), *n* = 57. Non-luminal breast cancer (**E–H**). N(−), *n* = 34; N(+), *n* = 8. *Statistically significant difference. N(−); negative ALN metastasis, N(+); positive ALN metastasis, SUVmax; maximum standardized uptake value, SAT; subcutaneous adipose tissue, V/S ratio; VAT SUVmax/SAT SUVmax. (**A–H**); *p*-values were determined by using Mann-Whitney *U* test.

In luminal breast cancer patients, as shown in Fig. 1, PET color-map showed significantly higher SUVs in the VAT region with ALN metastasis than negative ALN metastasis. Consistent with this finding, the patients with ALN metastasis showed significantly higher VAT SUVmax (0.4 ± 0.08 vs. 0.36 ± 0.09, *p* = 0.008) and V/S ratio (1.76 ± 0.38 vs. 1.55 ± 0.28, *p* < 0.001, Fig. 2A,C) than the patients without ALN metastasis. Furthermore, tumor SUVmax was also higher in ALN metastasis group than that in negative ALN metastasis group (3.62 ± 2.03 vs. 2.73 ± 1.92, *p* = 0.004, Fig. 2D). There was no statistically significant difference in SAT SUVmax between the two groups (Fig. 2B).

In non-luminal breast cancer patients, unlike to luminal breast cancer, there was no significant difference between the VAT SUVmax, V/S ratio, SAT SUVmax, and tumor SUVmax of ALN metastasis and negative ALN metastasis group (Fig. 2E–H).

### VAT metabolic activity in luminal A and B breast cancer

**Figure 3:**
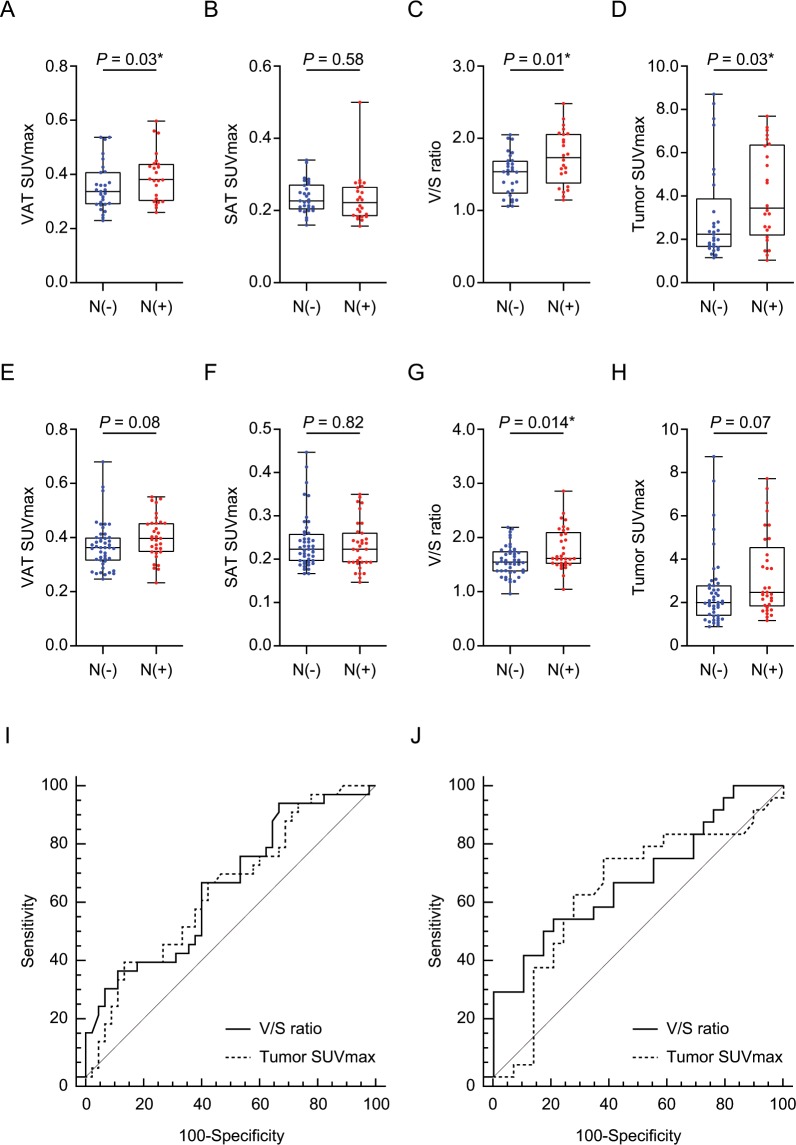
Comparison of (**A,E**) VAT SUVmax, (**B,F**) SAT SUVmax, (**C,G**) V/S ratio, and (**D,H**) Tumor SUVmax according to the ALN metastasis status in postmenopausal luminal A and B breast cancer patients. (**I,J**) Receiver operating characteristic (ROC) curve analysis for the prediction of ALN metastasis. Luminal A breast cancer (**A–D**, and **I**). N(−), *n* = 45; N(+), *n* = 33. Luminal B breast cancer (**E–H**, and **J**). N(−), *n* = 29, *n* =24. * Statistically significant difference. (**A–H**); *p*-values were determined by using Mann-Whitney *U* test.

In luminal A breast cancer patients, the patients with ALN metastasis exhibited significantly higher VAT SUVmax, V/S ratio, and tumor SUVmax (0.4 ± 0.08 vs. 0.37 ± 0.09, *p* = 0.03, 1.78 ± 0.39 vs. 1.57 ± 0.28, *p* = 0.01, and 3.26 ± 1.86 vs. 2.48 ± 1.66, *p* = 0.03; Fig. 3A,C,D, respectively) than the patients without ALN metastasis. There was no statistically significant difference in SAT SUVmax between the two groups, as expected (Fig. 3B). As shown in Fig. 3I, the optimal cut-off value of V/S ratio for prediction of ALN metastasis was 1.4 with a sensitivity of 93.9% and a specificity of 33.3%. Area under the curve (AUC) was 0.656 (95% confidence interval 0.54–0.76; standard error 0.06; *p* = 0.014). Considering the tumor SUVmax, the optimal cut-off value of tumor SUVmax for ALN metastasis was 3.56 with a sensitivity of 39.4% and a specificity of 86.7%. AUC was 0.644 (95% confidence interval 0.53–0.75; standard error 0.06; *p* = 0.022). There was no significant difference of AUC between V/S ratio and tumor SUVmax for the diagnosis of ALN metastasis (*p* = 0.911).

In luminal B breast cancer patients, the patients with ALN metastasis also exhibited significantly higher V/S ratio than the patients without ALN metastasis (1.74 ± 0.38 vs. 1.5 ± 0.29, *p* < 0.014, Fig. 3G). VAT SUVmax and tumor SUVmax were increased in ALN metastasis group than those in negative ALN metastasis group with marginal significance (0.39 ± 0.09 vs. 0.35 ± 0.09, *p* = 0.08 and 4.12 ± 2.19 vs. 3.11 ± 2.23, *p* = 0.07; Fig. 3E,H, respectively). SAT SUVmax showed no significant difference between the two groups. The optimal cut-off values of V/S ratio and tumor SUVmax for ALN metastasis were 1.69 and 2.41, respectively (Fig. 3J). For the optimal cut-off value of V/S ratio, the sensitivity and specificity were 54.2% and 79.3%, respectively with an AUC of 0.682 (95% confidence interval 0.54–0.8; standard error 0.08; *p* = 0.015). For the optimal cut-off value of tumor SUVmax, the sensitivity and specificity were 75% and 62%, respectively with an AUC of 0.643 (95% confidence interval 0.5–0.77; standard error 0.08; *p* = 0.077). There was no significant difference of AUC between V/S ratio and tumor SUVmax (*p* = 0.737).

### The effect of postmenopausal hormone replacement therapy on VAT metabolic activity

**Figure 4:**
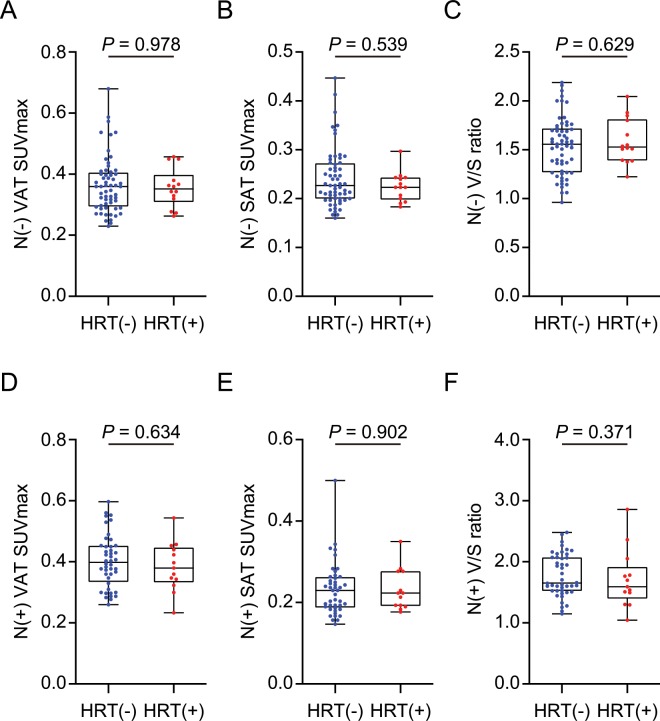
The effect of hormone replacement therapy (HRT) on (**A**) VAT SUVmax, (**B**) SAT SUVmax, and (**C**) V/S ratio in N(−) and (**D**) VAT SUVmax, (**E**) SAT SUVmax, and (**F**) V/S ratio in N(+) postmenopausal luminal breast cancer patients. (N−), *n* =p74, HRT(−), *n* = 60, HRT(+), *n* = 14; N(+), *n* = 57, HRT(−), *n* = 44, HRT(+), *n* = 13. HRT(−); patients without the use of HRT, HRT(+); patients with current HRT use. (**A–F**); *p*-values were determined by using Mann-Whitney *U* test.

As shown in Fig. 4, there was no statistically significant difference in VAT SUVmax, V/S ratio, and SAT SUVmax between current hormone replacement therapy (HRT) users and non-HRT use patients in both ALN metastasis and negative ALN metastasis group.

### Uni- and multivariable analyses

Univariable analysis showed that BMI, pathologic T stage, lymphovascular invasion, tumor SUVmax, and V/S ratio were significantly associated with ALN metastasis (Table 2). In the further multivariable analysis, lymphovascular invasion and V/S ratio were significantly associated with ALN metastasis and among the included variable, V/S ratio had the highest odds ratio for ALN metastasis.

**Table 2:** Uni- and multivariable analyses for prediction of axillary lymph node metastasis in postmenopausal luminal breast cancer patients.

Variable	Univariable analysis		Multivariable analysis	
	OR (95% CI)	*p*	OR (95% CI)	*p*
Age (continous)	0.985 (0.941–1.031)	0.512		
BMI (<25 kg/m^2^ vs ≥25 kg/m^2^)	2.259 (1.116–4.572)	0.024*	1.749 (0.749–4.083)	0.196
Histologic grade (1–2 vs 3)	1.386 (0.586–3.279)	0.457		
Pathologic T stage (T1 vs T2–T4)	4.279 (2.036–8.996)	<0.001*	1.828 (0.712–4.693)	0.21
Histology (ductal vs lobular and other)	0.723 (0.265–1.974)	0.527		
Lymphovascular invasion (negative vs positive)	6 (2.433–14.797)	<0.001*	4.57 (1.562–13.367)	0.006*
Luminal type (A vs B)	1.129 (0.559–2.28)	0.736		
Tumor SUVmax (regarding molecular subtype)	3.451 (1.651–7.215)	0.001*	2.38 (0.942–6.012)	0.067
V/S ratio (regarding molecular subtype)	3.573 (1.657–7.705)	0.001*	5.156 (2.041–13.028)	0.001*
Hormone replacement therapy (none vs current user)	1.266 (0.542–2.96)	0.586		

*BMI* body mass index, *SUVmax* maximum standardized uptake value, V/S ratio visceral adipose tissue SUVmax/subcutaneous adipose tissue SUVmax, *OR* odds ratio, *CI* confidence interval.

^*^Statistically significant difference.

### VAT metabolic activity reflects the systemic inflammation in luminal breast cancer patients

**Figure 5:**
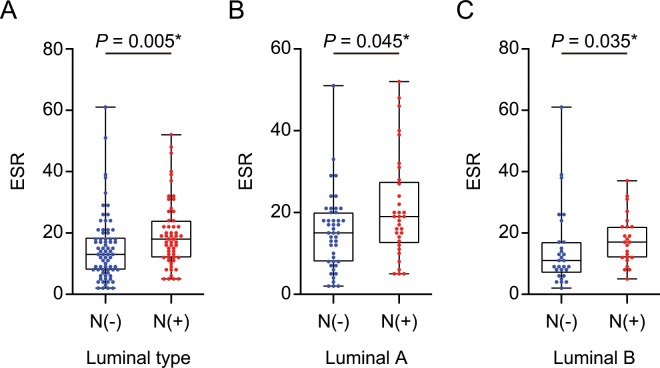
Comparison of erythrocyte sedimentation rate (ESR) according to the ALN metastasis status in (**A**) luminal type, (**B**) luminal A, and (**C**) luminal B breast cancer patients. (**A**) N(−), *n* = 74; N(+), *n* = 57, (**B**) N(−), *n* = 45; N(+), *n* = 33, (**C**) N(−), *n* = 29; N(+), *n* = 24. (**A–C**); *p*-values were determined by using Mann-Whitney *U* test.

In luminal breast cancer including luminal A and B, ESR, a surrogate marker for systemic inflammation^17^, was significantly higher in ALN metastasis patients than the patients without ALN metastasis (Fig. 5) and it was also significantly correlated with V/S ratio (Table 3). However, neither VAT SUVmax nor SAT SUVmax was correlated with ESR.

**Table 3:** Spearman correlation analysis between metabolic parameters of adipose tissue and the ESR in postmenopausal luminal breast cancer patients.

	VAT SUVmax		SAT SUVmax		V/S ratio	
	*r*	*p*	*r*	*p*	*r*	*p*
ESR (Luminal type)	0.085	0.337	−0.15	0.086	0.275	0.001*
ESR (Luminal A)	0.149	0.192	−0.106	0.354	0.293	0.009*
ESR (Luminal B)	0.039	0.78	−0.239	0.085	0.303	0.028*

ESR erythrocyte sedimentation rate, SUVmax maximum standardized uptake value, VAT visceral adipose tissue, SAT subcutaneous adipose tissue, V/S ratio VAT SUVmax/SAT SUVmax.

*Statistically significant difference.

## Discussion

To the best of our knowledge, this is the first study reporting the association between VAT metabolic activity and tumor aggressiveness in postmenopausal luminal breast cancer patients using ^18^F-FDG PET/CT. In this study, we clearly identified that the VAT metabolic activity assessed by preoperative ^18^F-FDG PET/CT was significantly associated in patients with ALN metastasis compared to patients with negative ALN metastasis, and was correlated with systemic inflammation in postmenopausal luminal breast cancer.

To date, extensive studies regarding the effect of obesity on breast cancer risk or cancer-related outcome, use BMI as a surrogate marker for obesity^4,5^. Although BMI is readily available marker of obesity, it is a crude measurement of obesity and does not fully reflect the dysfunctional VAT activity, a key pathophysiological mechanism which leads to the metabolic and clinical consequences of obesity^18,19^. In contrast, ^18^F-FDG uptake reflects the activity of M1 macrophage which is a predominant inflammatory cell type in dysfunctional VAT and secretes pro-inflammatory cytokines thereby enhancing tumor aggressiveness^5,15^. In the present study, we found that the VAT metabolic activity, which was defined as V/S ratio and assessed by ^18^F-FDG PET/CT, was elevated in ALN metastasis patients and showed a stronger association with ALN metastasis than BMI. Thus, we believe that the V/S ratio could serve as a surrogate marker for dysfunctional VAT related tumor aggressiveness in addition to BMI, especially in postmenopausal luminal breast cancer patients.

Unlike luminal breast cancer, V/S ratio exhibited no significant difference between the ALN metastasis and negative ALN metastasis group in postmenopausal non-luminal breast cancer patients. This finding is consistent with previous studies that revealed non-significant relationship between obesity and tumor aggressiveness in postmenopausal non-luminal breast cancer^4,5^. Although the exact mechanism underlying this observation is not clearly known yet, this result might be attributed to the different response to the estrogen produced in response to dysfunctional VAT in postmenopausal woman, between luminal and non-luminal breast cancer.

Some observational studies have reported that the use of HRT in postmenopausal woman might attenuate the obesity-related tumorigenic effect in luminal breast cancer patients^20,21^. Otherwise, several other studies have shown that there was no effect modification of the obesity-breast cancer relationship by postmenopausal HRT use^4,22,23^, and in this study we found that the current HRT use had no significant effect in VAT metabolic activity across ALN metastasis status in postmenopausal luminal breast cancer patients. This discrepancy may be due to the study’s reliance on self-reported HRT history and the use of different surrogate marker for the assessment of obesity-breast cancer relationship. As the obesity-inflammation-aromatase axis plays crucial roles in tumorigenesis in postmenopausal luminal breast cancer^5^, further study is warranted to elucidate the detailed mechanism on the effect of postmenopausal HRT use on the clinical outcome of luminal breast cancer.

Regarding ^18^F-FDG PET/CT for evaluation of ALN metastasis, one of the most commonly used parameters is the tumor SUVmax^24,25^. In this study, we found that both V/S ratio and tumor SUVmax measured by preoperative ^18^F-FDG PET/CT were significantly associated with ALN metastasis and the V/S ratio showed better association with ALN metastasis than the tumor SUVmax in luminal breast cancer patients (Table 2). Consistent with our results, Kim *et al*.^25^ report that the tumor SUVmax is effective to predict ALN metastasis and could be used as a prognostic biomarker in luminal breast cancer patients. Thus, based on our results, we believe that V/S ratio can used as a prognostic biomarker in luminal breast cancer patients that may improve accurate prediction of ALN metastasis in addition to the tumor SUVmax.

Recently, several large prospective studies and meta-analyses have reported that increased physical activity after a diagnosis of breast cancer lowers breast cancer related- and all-cause mortality in postmenopausal luminal breast cancer patients^26–28^. Furthermore, ongoing, large, randomized clinical trials have aimed to determine whether lifestyle modification such as diet and exercise can improve the clinical outcomes of breast cancer including postmenopausal luminal breast cancer^5^. Considering that VAT dysfunction is a causal mechanism which contribute to obesity-driven breast cancer, it is conceivable that V/S ratio assessed by ^18^F-FDG PET/CT might be employed as a potential biomarker of obesity-associated tumor aggressiveness and could be useful to evaluate the effect of therapeutic interventions against obesity in postmenopausal luminal breast cancer patients.

Several previous studies report that VAT has more inflammatory cells infiltration with elevated pro-inflammatory cytokines than SAT thereby contributing significantly to the increased systemic inflammatory condition which eventually leads to an increased risk of cardiovascular disease (CVD)^29–31^. Furthermore, recently, Kaess *et al*.^32^ report that relative fat depot ratio which defined as VAT/SAT ratio is correlated with CVD risk above and beyond VAT. Consistent with the results of these studies, we found that VAT SUVmax was higher than SAT SUVmax and V/S ratio was more highly correlated with systemic inflammation than VAT SUVmax itself. Thus, we believe that V/S ratio can be a unique inflammatory biomarker associated with the dysfunctional VAT.

Whereas our data suggest an association between V/S ratio and obesity-driven systemic inflammation in breast cancer patients, the detailed underlying mechanism remains to be determined. Furthermore, it is not clear how V/S ratio works in the patients with insulin resistance or diabetes. Insulin resistance or diabetes attenuates the normal regulatory response of VAT to insulin via down-regulation of mammalian target of rapamycin complex 2 (mTORC2) and phosphoinositide 3-kinase (PI3K) thereby promoting macrophage infiltration to VAT which leads to boost VAT inflammation^33,34^. Thus, it is anticipated that V/S ratio would be higher and would exhibit strong correlation with systemic inflammation in the patients with insulin resistance or diabetes than in those without such diseases. However, future investigation is warranted to elucidate the role of V/S ratio in the patients with insulin resistance or diabetes and the detailed underlying mechanism.

This study has several limitations. First, this was a retrospective study conducted at single center which could induce selection bias. A further prospective large study is warranted to validate the findings in this study. Second, although ^18^F-FDG PET/CT is a well-established method for the evaluation of VAT metabolic activity^12–15^, we could not obtain the tissue sample from the VAT for histopathological analysis. Finally, we were unable to control all possible factors that could affect FDG uptake, such as insulin and plasma glucose levels of patients, nor the image acquisition time after FDG injection.

Taken together, we provide strong evidence that VAT metabolic activity, defined as V/S ratio and evaluated by preoperative ^18^F-FDG PET/CT, could predict ALN metastasis in postmenopausal luminal breast cancer patients. As V/S ratio is a simple non-invasive method, it can be readily applied to daily clinical practice and can support the accurate staging which may helpful to improve the prognosis of postmenopausal luminal breast cancer patients. Furthermore, it can also be used as a surrogate marker of obesity-driven systemic inflammation.

## Methods

### Patients

Korean postmenopausal patients with newly diagnosed breast cancer who underwent preoperative ^18^F-FDG PET/CT from January 2013 to January 2015 were enrolled in this retrospective study. Patients with other cancer history, who received neoadjuvant chemotherapy before surgery, who had previously underwent abdominal surgery, who had active fever, any symptom of infection, or active systemic inflammatory comorbidity prior to surgery, or patients treated with any medication that could affect systemic inflammation within six months before taking preoperative ^18^F-FDG PET/CT were excluded. A total of 173 patients who had sentinel lymph node biopsy, ALN dissection, or both, with surgical resection of primary breast cancer were included. Final pathological results were confirmed through the examination of surgical specimens. Erythrocyte sedimentation rate (ESR) was measured using the modified Westergren method.

This study conformed to the guidelines of the Declaration of Helsinki and approved by the Institutional Review Board at Korea University Anam Hospital (Approval No. 2018AN0191). For this retrospective study, formal consent was waived by the Institutional Review Board.

### ^18^F-FDG PET/CT protocol

After overnight fasting, all patients received 5.29 MBq/kg of ^18^F-FDG. After 1 h of ^18^F-FDG injection, ^18^F-FDG PET/CT images were acquired using a commercially available PET/CT scanner (Gemini TF, Philips Medical Systems, Cleveland, OH, USA), which is a time-of-flight capable and fully three-dimensional scanner composed of a lutetium-yttrium oxyorthosilicate full-ring PET scanner and 16-slice helical CT scanner. The PET/CT scan covered the region from the skull vertex to the proximal thighs. The CT scan (120 kVp, 50 mA, 4 mm slice thickness) was carried out for attenuation correction at first, then followed by the PET scan. The PET scan (18 cm axial field of view with a 4.4 mm spatial resolution) was performed for 9 bed positions at 1 min per bed position. The PET images were reconstructed by iterative algorithm (three-dimensional row-action maximum likelihood algorithm) using attenuation correction map from the CT scan.

### Image analysis

All Images were evaluated by two experienced nuclear medicine physicians (KP and SK) using a commercially available workstation (Extended Brilliance Workspace version 3.5, Philips Healthcare, Eindhoven, Netherlands). The physicians were blinded to the clinical data of the patients.

For assessment of metabolic activity in the adipose tissue, VAT and subcutaneous adipose tissue (SAT) were identified in CT images using predefined Hounsfield units (range −70 to −110), as previously described^12–15^. Next, region of interest (ROI) was placed on each VAT and SAT region, and the standardized uptake (SUV) was calculated as follows:

$$SUV=Traceractivity\,(ROI)\,(MBq/mL)/Injected\,dose\,(MBq)/Total\,body\,weight\,(g)$$

For the assessment of VAT activity (see Supplementary Fig. S1), ROIs were selected on three consecutive slices of retroperitoneal VAT area, between the fourth and fifth lumbar vertebrae, and manually adjusted to exclude overspill ^18^F-FDG uptake in the intestine, vessel, and/or muscle, as previously described^13–15,35^. The highest SUVs from the three consecutive ROIs were acquired and the average defined as the VAT SUVmax. For the assessment of SAT activity, three consecutive ROIs were drawn on the anterolateral abdominal wall or both buttock area, as previously described^14,15^. The highest SUVs from the three consecutive ROIs were acquired and the average was determined as the SAT SUVmax. VAT metabolic activity was defined as VAT/SAT (V/S) ratio as follows:

$$V/S\,ratio=VAT\,SUVmax/SAT\,SUVmax$$

For each patient, ROIs were also manually drawn over the primary tumor and the tumor SUVmax was determined as the highest SUV within the ROI.

### BMI measurement and the classification of HRT user

BMI was calculated as weight/height squared (kg/m^2^). HRT user was defined as the patients who took oral conjugated equine estrogen and/or medroxyprogesterone acetate^4^ and subgroups were determined by the self-report of HRT use. Patients with current HT use were categorized as “current”, and all others were categorized as “none”.

### Pathology

All pathological evaluations were carried out on surgical specimens. Histologic grade was performed using the Nottingam Grading System including tubule formation, pleomorphism, and mitotic counts (grade 1: well-differentiated; grade 2: moderately differentiated; grade 3: poorly differentiated)^36^. Pathologic T stage was assessed according to the American Joint Committee on Cancer (AJCC) 8^th^ edition^37^. Histology was classified as invasive ductal, invasive lobular, and others by morphological analysis of tumor tissue. Lymphovascular invasion was defined as the tumor cell present within a definite vessel and/or lymphatics in the breast tissue surround tumor^38^.

### Classification of breast cancer molecular subtypes

Estrogen receptor (ER) and progesterone receptor (PR) status were defined as positive when the 10% of tumor nuclei or more were stained positively. Human epidermal growth factor receptor 2 (HER2) positive was defined as tumors with staining of 3+ (strong complete membranous staining in more than 10% of tumor cells). Ki-67 status was expressed according to the percentage of positive cells using a cut-off value of 20% of positive cells^39^.

According to the 2013 St. Gallen Consensus meeting^39^, molecular subtypes of breast cancer were defined as follows: Luminal A (ER and/or PR-positive, HER2-negative, and Ki-67 < 20%); Luminal B which include Luminal B: HER2-negative (ER and/or PR-positive, HER2-negative, and Ki-67 ≥ 20%) and Luminal B: HER2-positive (ER and/or PR-positive, HER2-positive, and any Ki-67 status); HER2-overexpression (ER-negative, PR-negative, and HER2-positive); and Triple-negative (ER-negative, PR-negative, and HER2-negative).

### Statistical analysis

The chi-squared (χ^2^) test or Fisher’s exact test was used for categorical variables. For continuous variables, Shapiro-Wilk test was used to assess the normal distribution of variables. Student’s *t*-test and Mann-Whitney *U* test were used for parametric and non-parametric analyses, respectively. Receiver-operating characteristic (ROC) curve analysis, logistic regression analysis, and Spearman correlation analysis were also used as statistical methods. The optimal cut-off value was determined by selecting the point which was corresponding to maximum Youden index (sensitivity-[1-specificity]) on the ROC curve. SPSS software version 17.0 (SPSS Inc, Chicago, IL, USA) and MedCalc software version 18.1 (MedCalc, Mariakerke, Belgium) were employed for data analysis. A *p*-value of < 0.05 was considered statistically significant.

## Supplementary information

Supplementary Figure S1.

## Data Availability

The datasets used and/or analysed during the current study are available from the corresponding author on reasonable request.
