# Supplementary material for: Visceral fat metabolic activity evaluated by preoperative 18F-FDG PET/CT significantly affects axillary lymph node metastasis in postmenopausal luminal breast cancer
Source: Sci Rep. 2020 Jan 28;10:1348. doi: 10.1038/s41598-020-57937-4 (PMC6987196; doi:10.1038/s41598-020-57937-4)
Supplement: Supplementary file 1 — Supplementary Figure S1. [file 41598_2020_57937_MOESM1_ESM.docx]

**Supplementary Information**

**Visceral fat metabolic activity evaluated by preoperative ^18^F-FDG PET/CT significantly affects axillary lymph node metastasis in postmenopausal luminal breast cancer**

Kisoo Pahk^1, 2^, Chanmin Joung^2^, and Sungeun Kim^1*^

*^1^Department of Nuclear Medicine, Korea University Anam Hospital, Seoul, Republic of Korea*

*^2^Institute for Inflammation Control, Korea University, Seoul, Republic of Korea*

***Corresponding Author:**

Sungeun Kim, M.D., Ph.D.

Professor, Department of Nuclear Medicine

Korea University Anam Hospital

73, Inchon-ro, Seongbuk-Gu, Seoul 02841, Republic of Korea

Tel: 82-2-920-5540, Fax: 82-2-921-2971, E-mail: seiong@korea.ac.kr


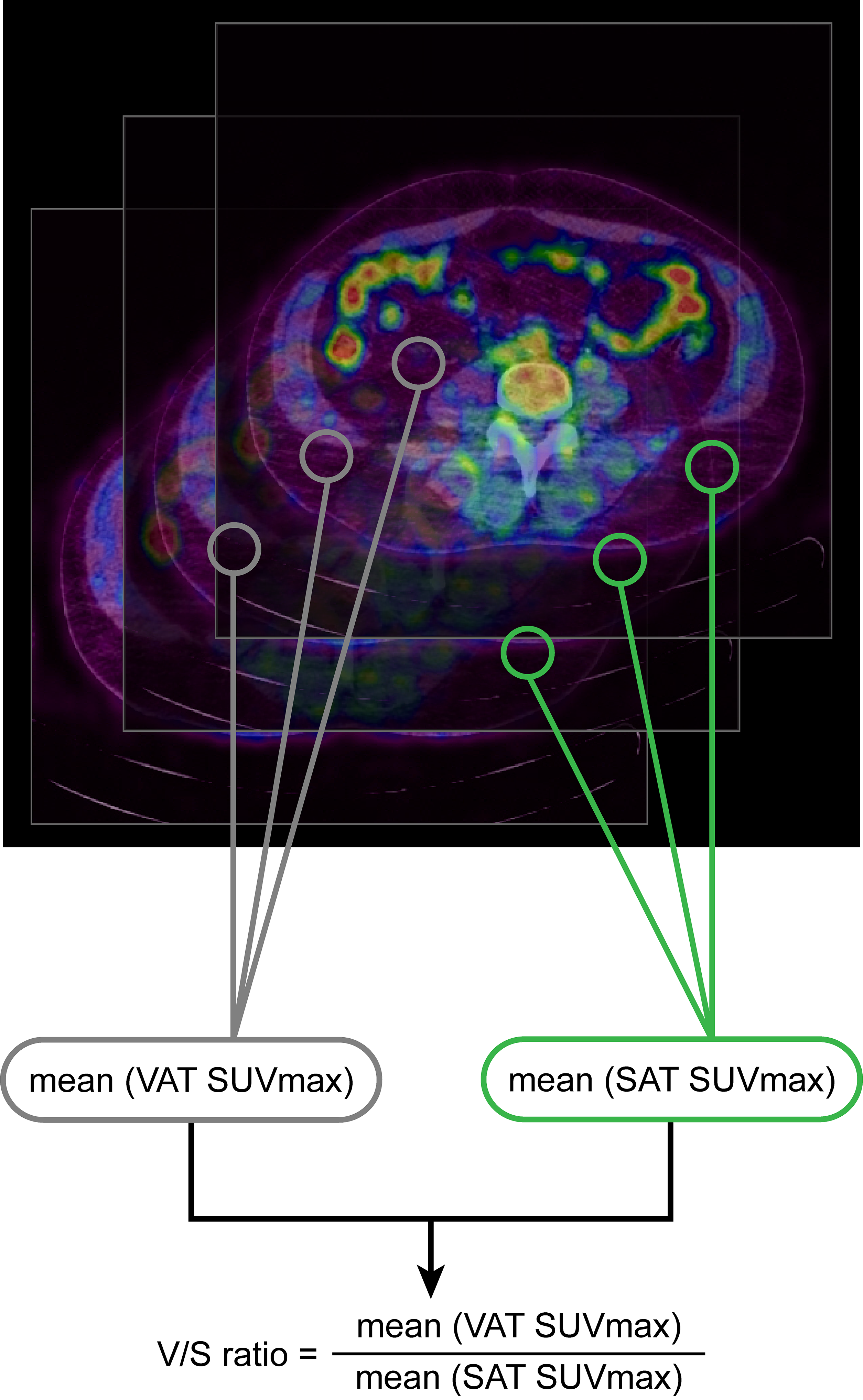


**Supplementary Figure S1.** Image analysis of VAT metabolic activity. Standard regions of ROIs were placed on VAT (grey) and SAT (green) on 3 consecutive slices. SUVmax was acquired from each ROI and SUVmax values across those slices were averaged. Afterward, VAT metabolic activity (V/S ratio) was calculated from mean VAT SUVmax divided by mean SAT SUVmax. VAT; visceral adipose tissue, ROI; region of interest, SAT; subcutaneous adipose tissue, SUVmax; maximum standardized uptake value, V/S ratio; VAT SUVmax/SAT SUVmax.
